# The involvement of orexin-1 receptors in modulation of feeding and anxiety-like behavior in rats with complete Freund’s adjuvant-induced temporomandibular joint disorder

**DOI:** 10.1007/s10266-024-01021-0

**Published:** 2025-01-23

**Authors:** Mojtaba Hosaini, Mehdi Abbasnejad, Razieh Kooshki, Saeed Esmaeili-Mahani, Maryam Raoof, Reyhaneh Naderi, Ghizlane Aarab, Frank Lobbezoo

**Affiliations:** 1 Department of Biology, Faculty of Sciences, Shahid Bahonar University of Kerman, Kerman, Iran; 2 Department of Biology, Faculty of Sciences, Lorestan University, Khorramabad, Iran; 3 Department of Orofacial Pain and Dysfunction, Academic Centre for Dentistry Amsterdam (ACTA), University of Amsterdam and Vrije Universiteit Amsterdam, Amsterdam, The Netherlands; 4 Department of Orofacial Pain and Jaw Function, Faculty of Odontology, Malmö University, Malmö, Sweden

**Keywords:** Temporomandibular disorders (TMDs), Orofacial pain, Food intake, Anxiety, Orexin 1 receptor (OX1R)

## Abstract

Orexin-A (OXA), a neuropeptide produced in the hypothalamus, is recognized for its role in modulating orofacial nociception and regulating feeding behaviors, as well as its impact on psychophysiological responses. This study investigated the role of orexin-1 receptors (OX1R) in modulating nociceptive behaviors induced by noxious stimulation of the temporomandibular joint (TMJ) and the associated changes in mood and feeding behaviors in rats with complete Freund’s adjuvant (CFA)-induced temporomandibular disorders (TMDs). Bilateral cannulation of the lateral ventricles was performed in rats. To induce nociception, CFA was injected unilaterally into the left TMJ of the rats. Nociceptive behaviors were assessed using the hot plate and tail flick tests, while anxiety-like behavior and food intake were evaluated using an elevated plus maze (EPM) and a food preference device, respectively. The results demonstrated a significant increase in nociceptive scores and anxiety-like behaviors, along with reductions in water and food consumption following CFA injection. However, post-treatment with OXA at concentrations of 50 and 100 pM/rat significantly decreased thermal nociceptive scores, alleviated anxiety-like behavior, and increased water and food intake. These beneficial effects were reversed when OXA was co-administered with SB-334867 (40 nM/rat), an OX1R antagonist. Collectively, our findings suggest that OX1R signaling plays a role in the modulation of anxiety-like behavior and abnormalities in food intake in CFA-treated rats. Understanding the involvement of OXA and its receptors in CFA-induced TMJ nociception and behavioral changes may pave the way for potential therapeutic interventions targeting OX1R signaling in the management of TMD-associated symptoms.

## Introduction

Temporomandibular disorders (TMDs) encompass a range of musculoskeletal conditions characterized by pain and dysfunction in the masticatory muscles, temporomandibular joint (TMJ), and related anatomical structures [1, 2]. TMDs manifest in both painful and non-painful forms, with the painful variants often involving TMJ and/or muscle pain [3]. A large population-based study utilizing the research diagnostic criteria for TMDs (RDC/TMD) identified a 36% prevalence of painful TMDs, including myalgia and arthralgia, among adults aged 20–49 years [4]. As the most prevalent type of non-odontogenic orofacial pain, painful TMDs are a major driver of treatment seeking, healthcare costs, and diminished quality of life in affected individuals [2, 5]. Despite their widespread impact, the neuronal pathways that process noxious sensory information from TMJ tissues are not well-defined. Evidence suggests that ascending projections from the TMJ and jaw muscles primarily terminate at the spinal trigeminal nuclear complex and upper cervical dorsal horn, with neurons responsive to TMJ injury projecting to higher brain centers involved in nociceptive processing, such as the ventrolateral periaqueductal gray (vlPAG), rostral ventromedial medulla (RVM), and posterior thalamus in male rats [6, 7].

Psychological distress, notably anxiety, has also been implicated in TMD pathogenesis [8–12], affecting pain perception and potentially heightening pain responses [13].

TMDs often result in restricted jaw movement and TMJ locking, adversely affecting mandibular opening, biting, and chewing abilities [14, 15], which can lead to changes in food consumption patterns, meal duration, and eating frequency [16]. Pain during mastication is frequently reported as a prominent clinical feature of TMDs [17], highlighting the close link between feeding behaviors and mastication-related discomfort [18, 19]. Thus, evaluating food intake can offer valuable insights into the severity of TMD symptoms.

Orexin A (OXA) and orexin-B (OXB) are orexigenic peptides produced by orexinergic neurons in the lateral hypothalamus, with extensive projections throughout the brain [20]. These orexins bind to G-protein coupled receptors, namely orexin-1 receptors (OX1R) and orexin-2 receptors (OX2R). OX1R exhibits high selectivity for OXA, while OX2R is nonselective for OXA or OXB [21]. Orexins are involved in regulating numerous physiological functions including feeding behavior, energy homeostasis, anxiety-like behavior, stress response, and nociception processing [22].

Orexigenic neurons extensively project to pain modulation regions such as the PAG, locus coeruleus, and paragigantocellularis lateralis (LPGI). Evidence suggests that OXA administration decreases sensory neuron firing and nociception responses [20, 23]. Specifically, OXA acts as a potent analgesic in behavioral studies [24, 25]. Additionally, OXA inhibits trigeminal nerve firing [26, 27] and reduce capsaicin-induced orofacial nociception in rats [28].

Orexin receptors (OXRs) are present in brain regions that regulate feeding, including the hypothalamic and mesolimbic areas, underscoring OXA’s role in food intake and metabolic response [29, 30]. Studies show that OXA influences feeding behaviors and activates neurons involved in feeding regulation [21, 31, 32]. Administration of OXA stimulates feeding and increases energy intake [21], while blockade of OXA through pretreatment with the OX1R antagonist SB-334867 leads to increased food consumption [33, 34].

The existing literature has addressed OXA’s involvement in modulating orofacial nociception [28, 35] and feeding behaviors [21, 31, 32]. On the other hand, various studies have utilized the complete Freund’s adjuvant (CFA)-treated TMJ as a model for orofacial pain. Studies have shown that tumor necrosis factor alpha (TNFα) is significantly increased in CFA-treated TMJ tissues and plays a critical role in CFA-induced inflammatory TMJ pain [36]. Additionally, TMDs have been linked to thermal and mechanical hypersensitivity [37].

Therefore, this study aims to investigate the role of OX1R in modulating feeding and anxiety-like behaviors in rats with CFA-induced TMJ noxious stimulation, employing methods such as food intake measurements, the elevated plus maze (EPM), hot-plate, and tail-flick tests. Understanding the impact of OXA signaling in CFA-induced TMJ nociception and feeding behaviors may offer potential therapeutic implications for managing TMD-associated symptoms.

## Materials and methods

### Animals

Forty-eight adult male Wistar rats, weighing 200–250 g (2–3 months), were included in this study. The rats were housed in a room maintained at a controlled temperature of 21 ± 2 °C with a 12-h light/12-h dark cycle and had ad libitum access to food and water. We followed ethical standards for the investigation of experimental pain in animals, and the study was approved by the Animal Experimentation Ethics Committee of the Kerman Neuroscience Research Center (EC/KNRC/95–26).

### Drugs

CFA was sourced from Sigma-Aldrich, USA, and diluted in normal saline (1:1). OXA from Tocris, USA, was dissolved in distilled water. SB334867, an OX1R antagonist from Tocris, USA, was dissolved in dimethyl sulfoxide (DMSO) and further diluted with normal saline to ensure optimal solvation. The final concentration of DMSO was maintained below 0.1%. All drugs were freshly prepared on each experimental day.

### Surgery

**Fig. 1:**
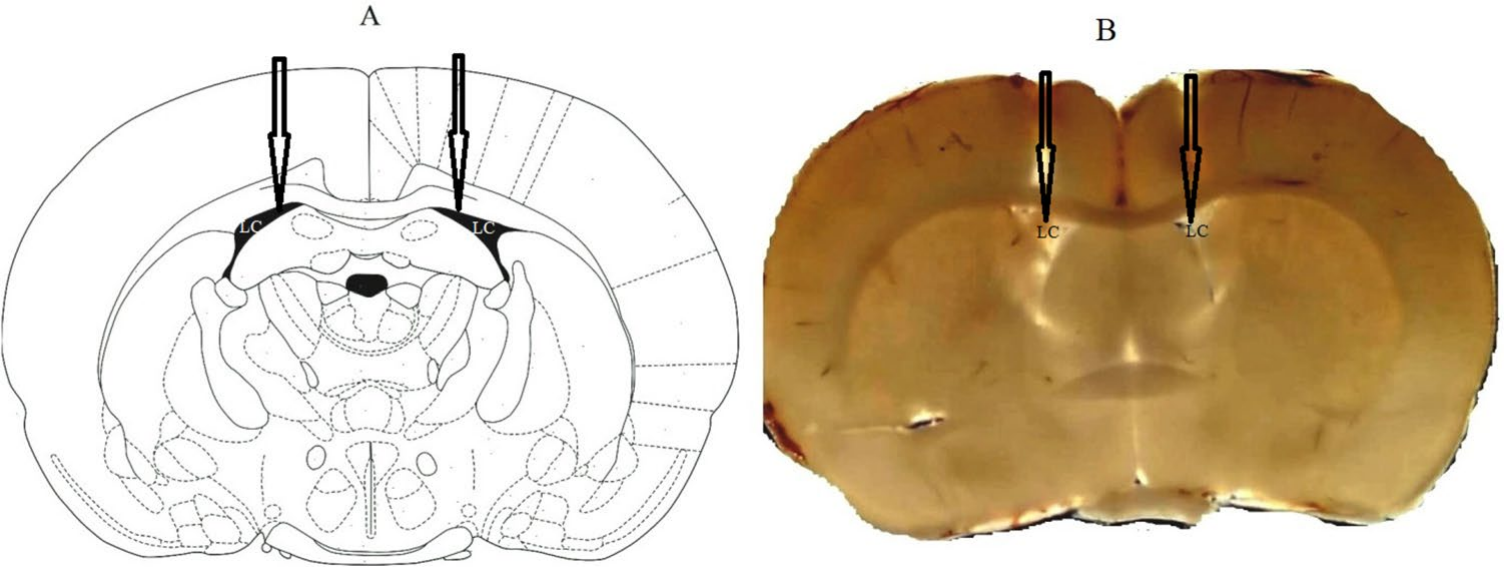
Descriptive lateral ventricles section replicated from the rat brain atlas of Paxinos and Watson (**A**). Illustrative brain coronal section displaying the injection site (**B**)

Animals were anesthetized with ketamine and xylazine (50 mg/kg and 5 mg/kg, respectively) and stereotaxically implanted with guide cannulas (Stoelting, USA). The 23-gauge stainless steel tubing guide cannulas were bilaterally implanted into the right and left ventricles at coordinates 1.6 mm posterior to the bregma, ± 0.8 mm lateral from the midline, and 3.4 mm deep from the cortical surface, based on the atlas of Paxinos and Watson. Two stainless steel screws and acrylic dental cement secured the guide cannulas to the skull, and the cannulas’ inlets were closed with a stylet. After surgery, the rats were housed individually and allowed a one-week recovery period before the drug injections and behavioral experiments [38]. The exact placement of each rat’s cannula was histologically verified at the end of the experiments. A representative brain coronal slice displaying the injection site is presented in Fig. 1.

### Microinjection

Drugs and their vehicles were administered using a 27-gauge needle connected via polyethylene tubing to a 1 μl Hamilton syringe. The injection needle was inserted 1 mm beyond the guide cannula tip. The needle remained in place for an additional minute after each infusion to ensure complete diffusion of the drugs. Rats with misplaced cannulas (four rats) were excluded from further behavioral analysis. Histological examination confirmed the correct placement of cannulas in the remaining rats.

### Induction of TMJ nociception

After a one-week recovery, the rats were anesthetized intraperitoneally with a mixture of ketamine and xylazine. Nociception was induced by a unilateral injection of 20 µL of CFA into the left TMJ using a 27-gauge needle. The anterosuperior portion of the zygomatic arch root was identified as the insertion point, following the method described by Kameoka and Xu. The needle was inserted percutaneously into the anterosuperior compartment of the TMJ, and the reagent was slowly administered. The time point of maximum inflammation and the head withdrawal reflex was set at 24 h after CFA administration [39].

### Histology processing

The rats were anesthetized by intraperitoneal administration of ketamine (100 mg/kg) and xylazine (10 mg/kg) and transcardially perfused with 100 mL of 0.9% saline followed by 500 mL of 4% paraformaldehyde in 0.01 M phosphate buffer. The TMJ tissues were then resected and fixed in 4% paraformaldehyde overnight. After decalcification in 10% EDTA in 0.1 M phosphate buffer for 14 days, the samples were dehydrated through an ascending series of ethanol, embedded in paraffin, and sectioned along their coronal axis at 7μ on a rotating microtome (Scilab Microsystems, ROTO-CUT200, London, UK). The sections were placed on gelatin-coated slides. Haematoxylin and eosin staining was used to examine the TMJ under a light microscope (Olympus BX41, Tokyo, Japan).

### Experimental groups

The rats were randomly divided into eight experimental groups, each consisting of six rats:

- Control group: no treatment.
- CFA-treated group (CFA): received CFA (100 μg/10 μL) into the left TMJ after cannulation.
- CFA plus distilled water (DW) group (CFA + DW): received DW (1 µL/i.c.v.) as the OXA vehicle 24 h after CFA injection.
- CFA plus DMSO group (CFA + DMSO): received DMSO as the SB-334867 vehicle 24 h after CFA injection.
- Three CFA plus OXA-treated groups (CFA + OXA): received OXA at concentrations of 25, 50, and 100 pM/rat/i.c.v., 24 h after CFA injection.
- CFA + OXA + SB-334867-treated group (CFA + SB + OXA): received SB-334867 (40 nM rat/i.c.v.) followed by OXA (50 pM rat/i.c.v.) 24 h after CFA injection.

**Fig. 2:**
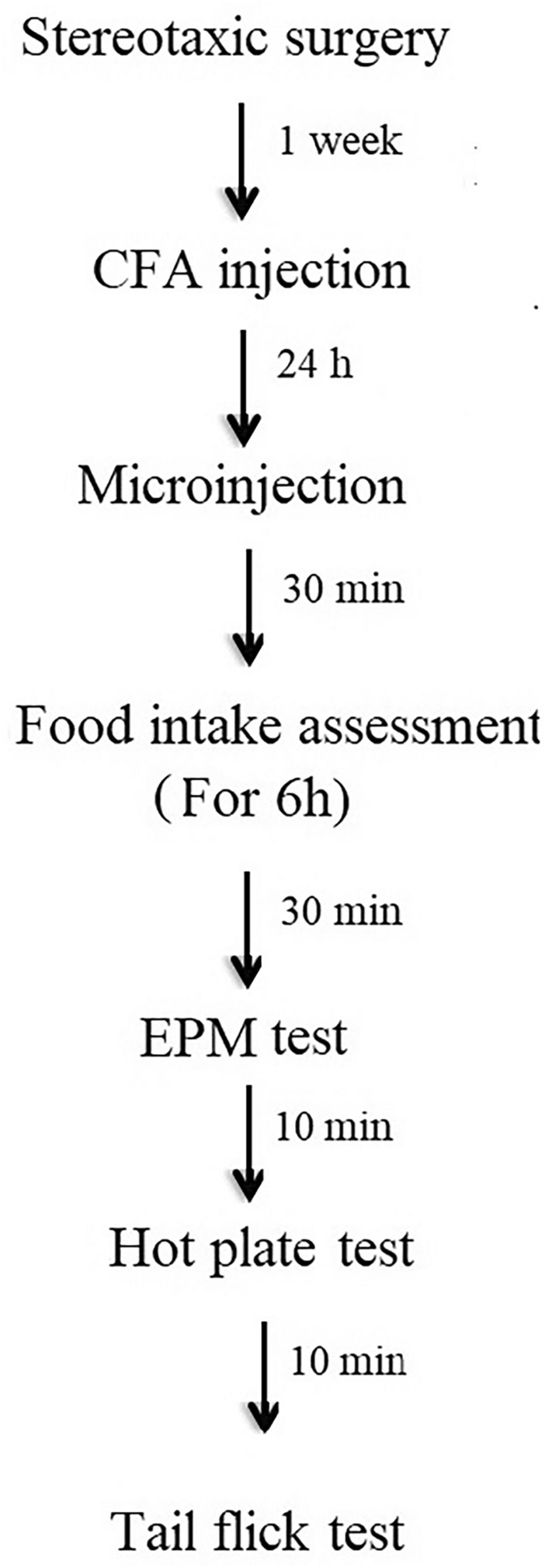
The experimental timeline

Experimenters were blinded during injections and behavioral evaluations. The experimental timeline is depicted in Fig. 2.

### Food intake box

To assess food intake behaviors 30 min after drug injections, an open field in the form of a sealed black Plexiglas box (60 cm long, 60 cm wide, and 30 cm high) was used. Rats were placed in the middle of the apparatus, equipped with load sensors to record and process the rats’ locations and their food and water consumption. A port on one internal side allowed free access to food and water. During the six-hour test period, the frequency of meals, the duration of time rats spent near food and water ports, and meal durations were recorded. The animals were fasted before the food intake examination.

### EPM test

The EPM test, a standard technique for measuring anxiety-like behavior in rodents, exploits rodents’ natural aversion to open spaces [40]. The maze, elevated 50 cm above the floor, included two open arms with 0.5-cm-high transparent Plexiglas ledges and two closed arms of the same size [60 cm] enclosed by 40-cm-high wooden walls. The four arms met in a central 5-cm-square area. Each rat was placed at the junction of the four arms, facing an open arm, and allowed to freely explore the maze for 5 min. The number of entries into the open arms and the time spent in both open and closed arms were recorded using a video tracking system and analyzed with Any-maze software.

### Hot-plate test

Rats were individually placed on a thermostatically controlled metal hot plate maintained at 55 ± 0.2 °C. A latency response, characterized by behaviors indicating discomfort such as flicking or licking of the hind and forepaws, or jumping to escape the heat, was recorded. The experiment was conducted three times for each rat, with a 1-min interval between trials. The maximum latency allowed before ending the test was 20 s to prevent tissue damage. Latency times within experimental groups were averaged and expressed as means.

### Tail flick test

A tail flick apparatus applied radiant heat to the base of the tail, 4–7 cm from the distal end. The tail-flick latency for each animal was determined over three trials, and the average was used as the thermal nociceptive threshold. A 1-min interval was allowed between each trial, and a cutoff time of 20 s was employed to prevent potential tissue damage.

### Statistical analysis

All data are expressed as the mean ± standard error of the mean (SEM). Parametric one-way analyses of variance (ANOVA) followed by Tukey’s post-hoc tests were conducted to assess nociceptive behaviors following TMJ noxious stimulation, and the results from hot plate, tail-flick, and EPM tests. The non-parametric Kruskal–Wallis test was used to analyze the data from the food intake task, while the Mann–Whitney U test compared differences in dependent variables between two independent groups. A P-value of less than 0.05 was considered statistically significant.

## Results

### CFA-Induced TMJ Inflammation

**Fig. 3:**
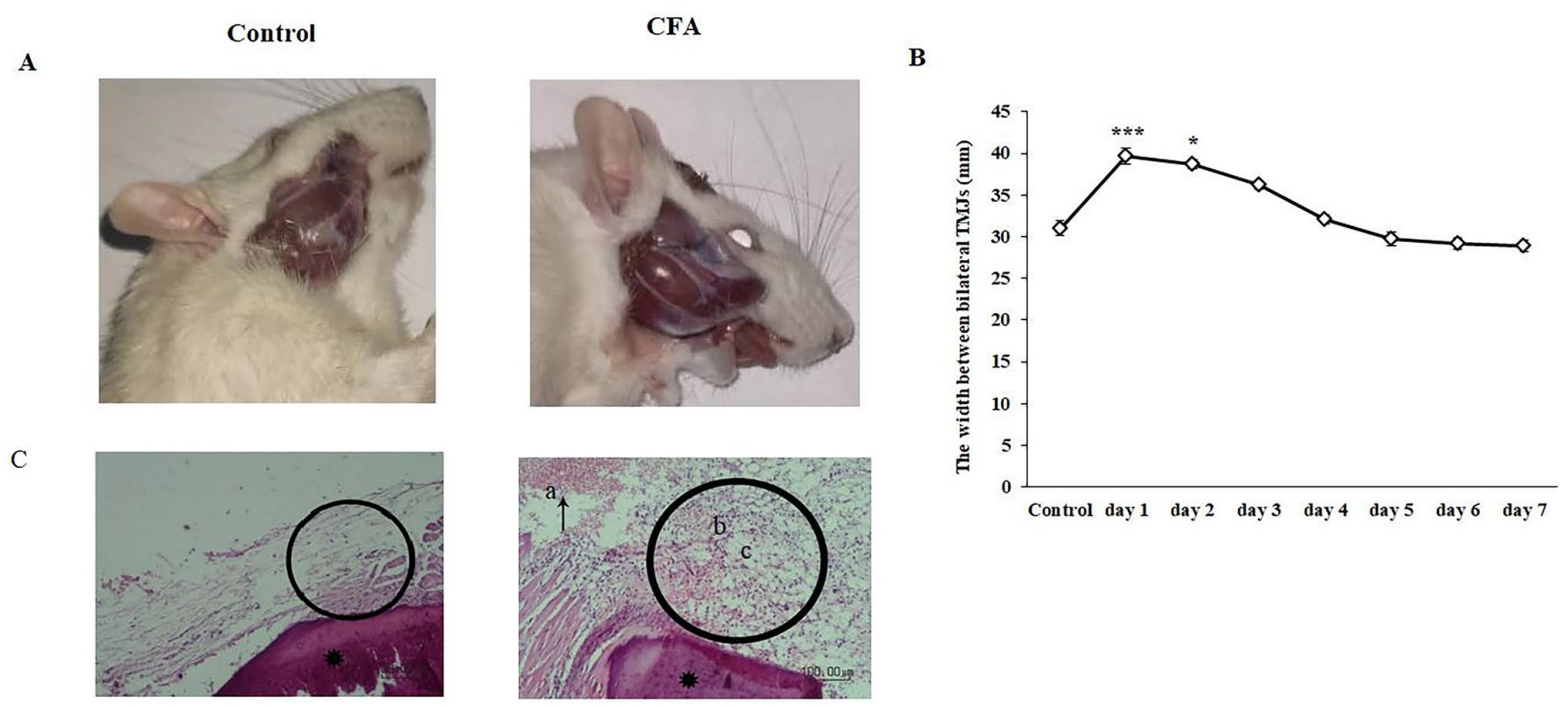
Apparent inflammation of TMJ area and chromodacryorrhea around the eyelid in CFA group as compared to the control (**A**); the linear head width between bilateral TMJs displayed remarkable increases at day 1 as well as day 2 (**B**); In comparison with the control group, hyperemia and perivascular hemorrhages (arrow/a); synovial cells apparent hyperplasia, marked inflammation (in the circle) and, lipid droplets (c), leukocyte (b) in the CFA group. Temporal bone (star)

Following CFA administration, the linear head width between bilateral TMJs exhibited significant increases on days 1 and 2 in CFA-treated rats compared to the control group (Fig. 3A). However, the width gradually decreased within seven days. Notable inflammation of the TMJ area and external signs of chromodacryorrhea around the eyelids were observed in CFA-treated rats (Fig. 3B). The synovial lining cells appeared hyperplastic with an abundant infiltration of mononucleated cells into the synovium. Numerous lipid droplets were detected in the inflamed synovium (Fig. 3C).

### Feeding behaviors

**Fig. 4:**
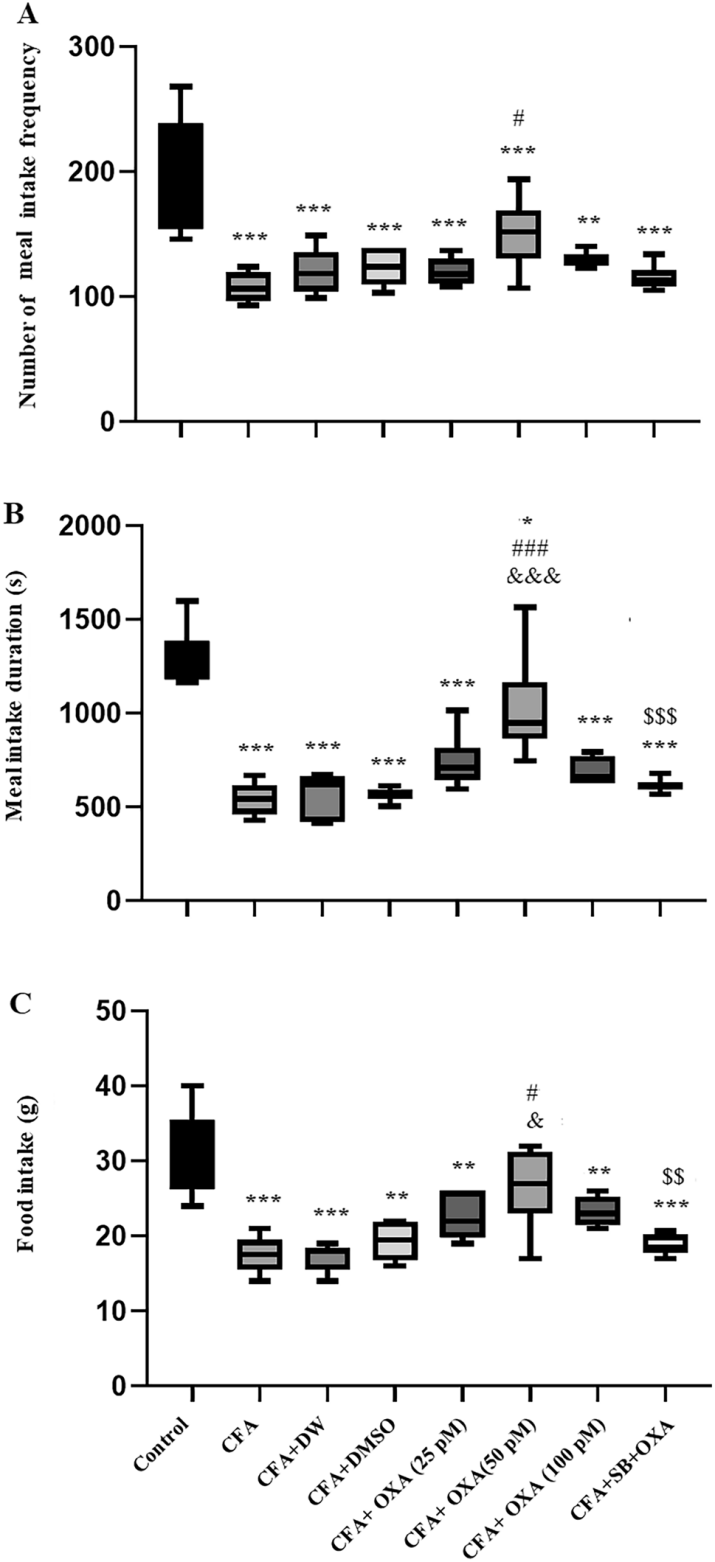
Effects of OXA, and SB + OXA administration into the lateral ventricles on meal duration (**A**), meal frequency (**B**), and food intake (**C**) in CFA-treated rats. The data in each graph are represented as mean ± SEM values. *CFA* complete Freund’s adjuvant, *OXA* orexin-A, *SB* SB-334768. *P < 0.05, **P < 0.01 and ***P < 0.001 versus control; ^#^P < 0.05, ^##^P < 0.01 and ^###^P < 0.001 versus CFA; ^&^P < 0.05, ^&&&^P < 0.001 versus CFA + DW, and CFA + DMSO, ^$$^P < 0.01 and.^$$$^P < 0.001 versus CFA + SB + OXA (50 pM)

Significant differences in meal duration [F(7, 47) = 22.159, P = 0.0001] (Fig. 4A) and meal frequency [F(7, 47) = 16.644, P = 0.0001] (Fig. 4B) were observed among the experimental groups. Both meal duration and meal frequency significantly decreased in the CFA and CFA plus vehicle (DW or DMSO)-treated groups compared to the control group. However, treatment with OXA (50 pM) significantly increased meal duration and the number of meals per day compared to the CFA group. Additionally, significant differences were observed in food intake [F (7, 47) = 22.159, P = 0.0001] among the experimental groups. Food intake in rats treated with CFA, CFA+DW, and CFA+DMSO was significantly decreased compared to the control group (P < 0.001). Conversely, orexin-A (50 pM) infusion significantly increased food intake compared to the CFA-treated group (P < 0.01) (Fig. 4C). Pretreatment administration of SB 334867 (40 nM/rat) inhibited orexin-A (50 pM/rat) potential to increase feeding-related behavior in CFA-treated rats.

**Fig. 5:**
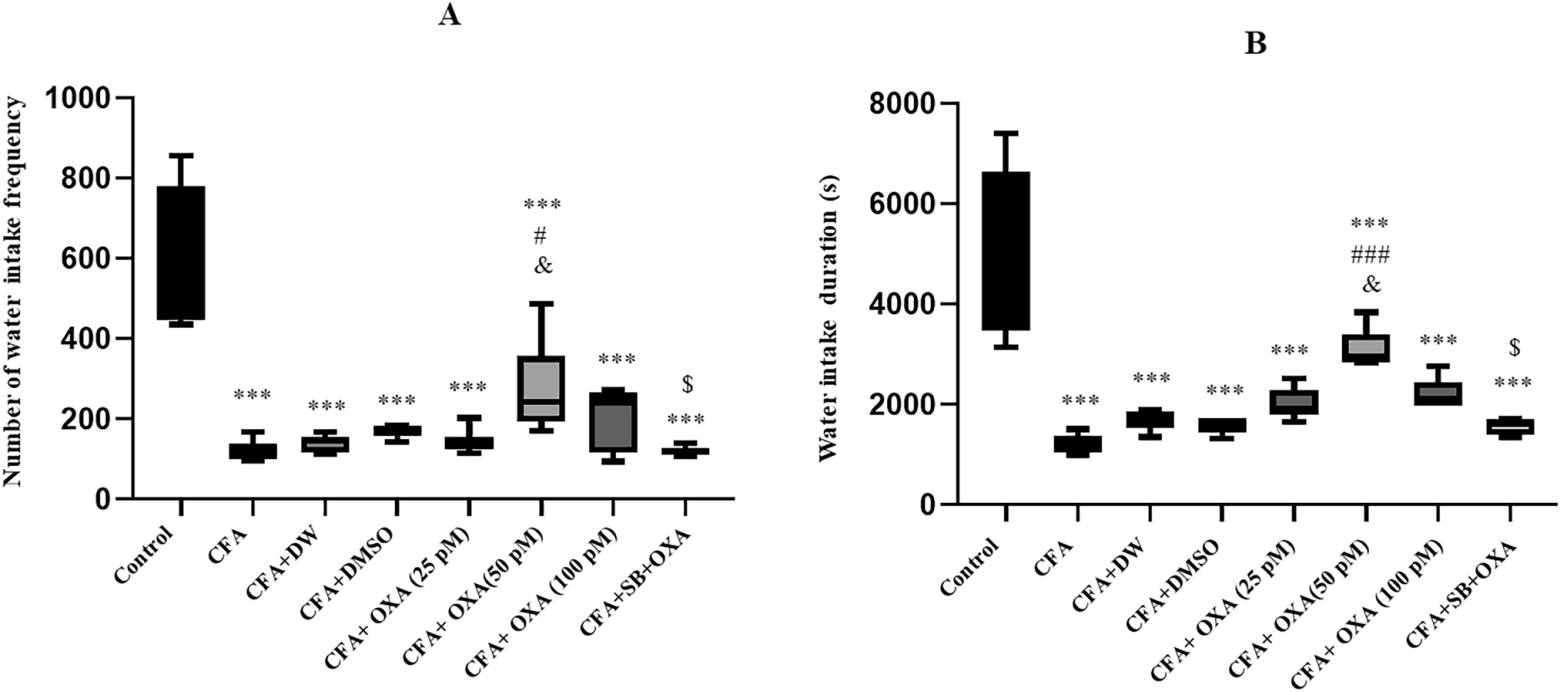
Effects of OXA and SB + OXA administration into lateral ventricles on water intake duration (**A**) and water intake frequency (**B**) in CFA-treated rats. The data in each graph are represented as mean ± SEM values. *CFA* complete Freund’s adjuvant, *OXA* orexin-A, *SB* SB-334768. ***P < 0.001 versus control; ^#^P < 0.05 versus CFA; ^&^P < 0.05 versus CFA + DW, and CFA + DMSO, ^$^P < 0.05 versus CFA + SB + OXA (50 pM)

Water intake duration [F(7, 47) = 20.990, P = 0.0001] (Fig. 5A) and water intake frequency [F(7, 47) = 23.810, P = 0.0001] (Fig. 5B) also showed significant differences among the experimental groups. Both parameters significantly decreased in the CFA and CFA plus vehicle groups compared to the control group (P < 0.001). However, OXA (50 pM) administration increased water intake duration (P < 0.01) and frequency (P < 0.05) compared to CFA-treated rats.

### Anxiety-like behavior

**Fig. 6:**
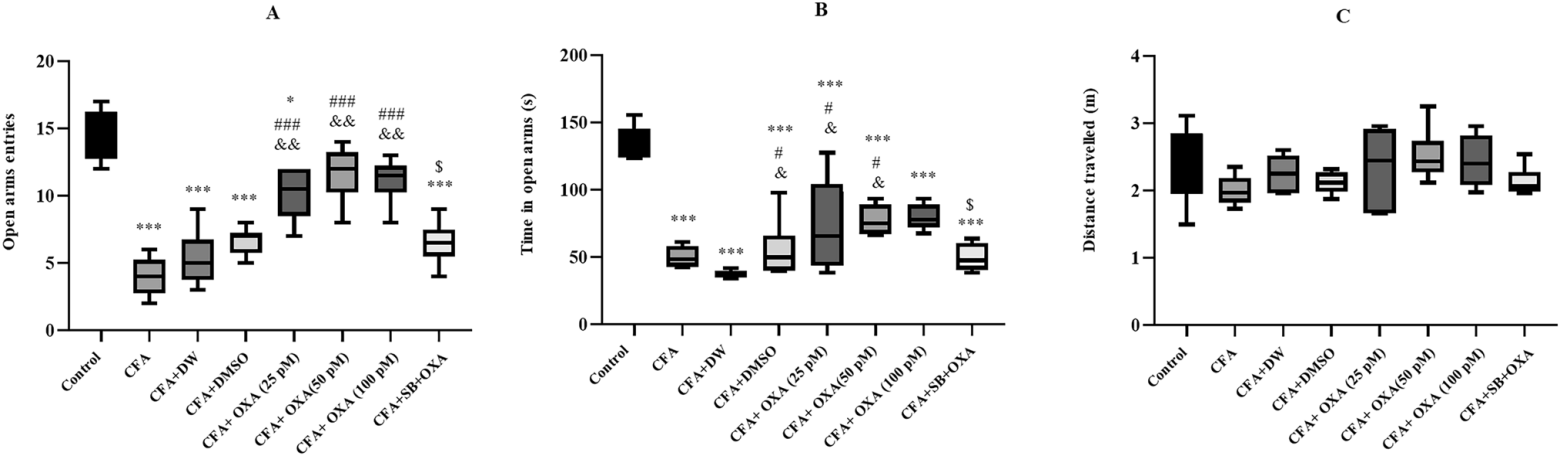
Effects of OXA and SB + OXA administration into lateral ventricles on anxiety-like behavior in CFA-treated rats. The data in each graph are represented as mean ± SEM values. *CFA* complete Freund’s adjuvant, *OXA* orexin-A, *SB* SB-334768. *P < 0.05 and ***P < 0.001 versus control; ^#^P < 0.05 and ^###^P < 0.001 versus CFA, ^$^P < 0.05 versus CFA + SB + OXA (50 pM)

Significant differences were observed in the time spent [F (7, 47) = 20.722, P = 0.0001] (Fig. 6A) and entries into [F (7, 47) = 16.644, P = 0.0001] the open arms of the EPM (Fig. 6B). Time spent in open arms and the number of entries in open arms were significantly increased in the CFA and CFA plus vehicle-treated groups compared to the control group (P < 0.001). However, microinjection of OXA at all doses (25, 50, 100 pM/rat) significantly increased the percentage of time spent (P < 0.05) and entries (P < 0.01) into the open arms compared to the CFA-treated group. Notably, pretreatment microinjections of SB 334867 (40 nM/rat) prevented OXA (100 pM/rat)-induced increases in the time spent and the number of entries into open arms. Total distance traveled in the maze showed no significant differences among experimental groups (Fig. 6C).

### Hot plate test

**Fig. 7:**
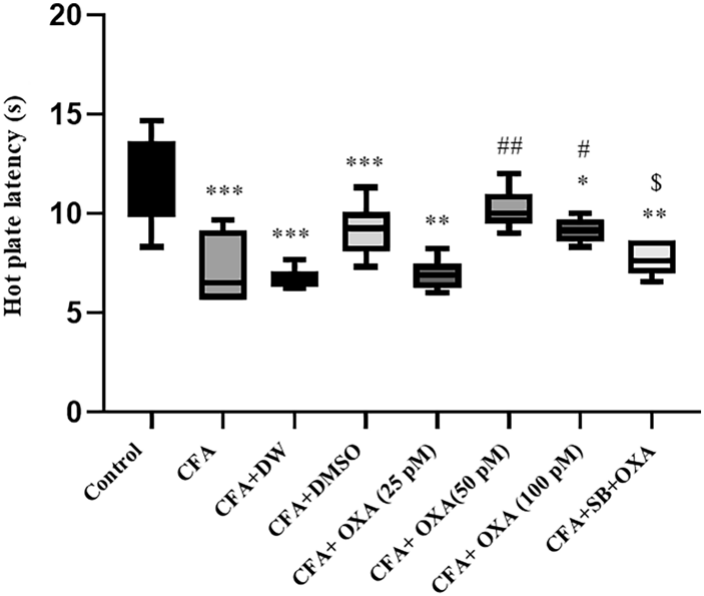
Effects of OXA and SB + OXA administration into the lateral ventricles on withdrawal latency values in CFA-treated rats in the hot plate test. The data in each graph are represented as mean ± SEM values. *CFA* complete Freund’s adjuvant, *OXA* orexin-A, *SB* SB-334768. *P < 0.05, **P < 0.01 and ***P < 0.001 versus control; ^#^P < 0.05 and ^##^P < 0.01 versus CFA, ^$^P < 0.05 versus CFA + SB + OXA (50 pM)

Significant alterations in hot plate latencies were observed between the groups [F (7, 47) = 16.644, P = 0.0001] (Fig. 7). The thermal nociceptive threshold was significantly decreased in the CFA and CFA plus vehicle-treated groups compared to the control group (P < 0.05). However, OXA administration at doses of 50 (P < 0.01) and 100 pM (P < 0.05), but not 25 pM, significantly increased the hot-plate latency compared to CFA-treated rats. Pretreatment with SB-334768 (40 nM/rat) significantly blocked the OXA-induced effects on hot plate latencies.

### Tail-flick test

**Fig. 8:**
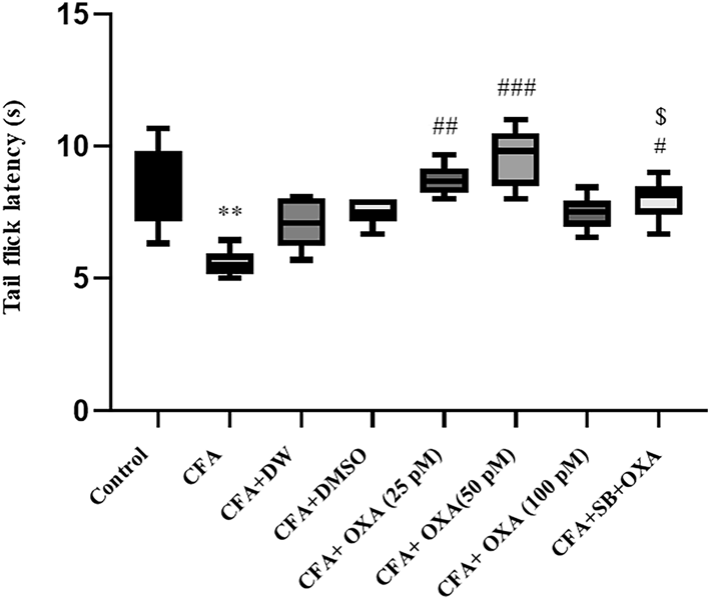
Effects of OXA and SB + OXA administration into lateral ventricles on withdrawal latency values in CFA-treated rats the tail flick test. The data in each graph are represented as mean ± SEM values. *CFA* complete Freund’s adjuvant, *OXA* orexin-A, *SB* SB-334768. **P < 0.01 versus control; ^#^P < 0.05, ^##^P < 0.01 and ^###^P < 0.001 versus CFA. *P < 0.05, **P < 0.01 and ***P < 0.001 versus control; ^#^P < 0.05 and ^##^P < 0.01 versus CFA. ^$^P < 0.05 versus CFA + SB + OXA (50 pM)

Significant differences in tail flick withdrawal latency were observed between the experimental groups [F(7, 47) = 19.855, P = 0.0001] (Fig. 8). The latency time was significantly decreased in CFA and CFA plus DW-treated rats compared to control animals (P < 0.01). OXA infusion at doses of 25 and 50 pM significantly increased withdrawal latency compared to CFA-treated rats (P < 0.01 and P < 0.001, respectively). Notably, the effects of OXA (50 pM) were significantly blocked through pretreatment with SB-334768 (40 nM/rat) (P < 0.05).

## Discussion

The primary objective of this study was to investigate the influence of OX1R on feeding behavior and anxiety-like responses in rats with CFA-induced TMJ noxious stimulation. The results revealed that administration of CFA heightened thermal nociceptive responses and increased anxiety-like behaviors, accompanied by reduced food and water intake. However, when OXA was administered into the lateral ventricles, it attenuated the CFA-induced effects. Remarkably, SB-334867 pretreatment reversed the effects of OXA on these behaviors, and it also increased food consumption in the TMJ-stimulated rat model. These findings suggest potential pathways through which the orexinergic system might modulate nociception associated with TMJ stimulation. While these results offer valuable insights, we must exercise caution when directly translating these findings to human TMD pain. Instead, they provide a foundation for further research into the mechanisms that might drive pain processes in TMDs, potentially guiding future therapeutic strategies.

In this study, the administration of CFA into the TMJ elicited nociceptive responses in both the tail-flick and hot plate tests. Intra-articular CFA injection has been suggested as a technique for inducing joint inflammation [41]. CFA-induced TMJ inflammation led to the activation of glial and immunological cells, which play crucial roles in the development of chronic pain conditions [42, 43]. Consistent with these result, previous studies on rodents have reported spinal hypealgesia in response to orofacial CFA injection. CFA induced contralateral orofacial hyperalgesia by stimulating the RVM and spinal trigeminal nuclei circuitry [44]. Furthermore, injection of CFA into the upper lip/whisker pad of the rat resulted in persistent thermal and mechanical hyperalgesia [45]. Additionally, CFA administration triggered mechanical allodynia and calcitonin gene-related peptide (CGRP) expressions in male rats [46].

In line with existing research, this study observed behavioral changes indicative of increased anxiety-like responses in rats subjected to CFA-induced TMJ noxious stimulation [9–12]. Orofacial pain, including TMD, has been consistently linked to negative psychological states, with anxiety being particularly prominent [13]. Previous studies have shown a close link between orofacial pain such as dental pulp nociception [47–49], headaches [50], trigeminal neuralgia, and TMDs [51]. Injuries to the trigeminal nerve have been associated with increased anxiety-like behaviors and facial pain [52]. Boleta-Ceranto et al. reported that orofacial nociceptive responses induced by formalin injection in the TMJ led to an elevation in anxiety levels in rats [47]. Additionally, individuals with chronic TMD have shown higher levels of anxiety [9]. These findings collectively highlight the complex interactions between nociceptive behaviors observed following CFA-induced TMJ noxious stimulation and anxiety-like responses in a rat model. This underscores the potential influence of orofacial nociception on emotional well-being, suggesting broader implications for understanding similar phenomena in TMDs.

Furthermore, our study revealed that CFA injection led to a reduction in both the duration and frequency of food and water intake, as well as a decrease in the overall amount of food consumption. This reduction in food intake has been shown to be closely linked to TMJ pain and could serve as a potential predictor of TMJ pain severity [53]. The restrictions in jaw movement associated with TMD may contribute to altered masticatory function, making food intake more challenging for affected individuals [14, 15]. Consequently, TMJ pain may manifest in altered eating patterns, including changes in food intake, meal duration, and frequency of consumption [16]. Nordahl and colleagues also observed a reduction in the daily food consumption of animals following CFA injection, leading them to consume fewer meals [54]. Notably, CFA-induced inflammation has been reported to decrease food intake and body weight in rats while simultaneously increasing corticosterone levels [55].

In our study, the intracerebroventricular (i.c.v.) injection of OXA demonstrated a significant inhibition of CFA-induced TMDs nociception and anxiety-like behaviors. Conversely, rats with TMJ noxious stimulation that received an OX1R antagonist exhibited a reduction in the mitigative effects on nociceptive and anxiety-like responses induced by OXA. Numerous investigations have provided evidence of an association between orexigenic peptides and the descending pain regulation circuits, highlighting their efficacy in reducing nociceptive responses [56]. The robust projections of orexigenic neurons to key pain transition pathways, including the spinal cord, brain stem, the spinal and trigeminal dorsal horns, and PAG, strongly support the involvement of orexin in the modulation of nociceptive pathways [20, 23]. Specifically, OXA has been demonstrated as a potent antinociceptive agent [24, 25], playing a critical role in modulating trigeminal pain transmission and effectively suppressing trigeminal nerve firing in rats [26, 27]. Kooshki et al. have provided evidence of the involvement of trigeminal nucleus caudalis OX1Rs in orofacial nociception transmission [28]. Moreover, studies have shown that OXA can alleviate capsaicin-induced trigeminal nociception by modulating cyclooxygenase 2(COX-2) expression in the trigeminal nucleus caudalis [57]. Infusion of OXA into the lateral ventricles of rats resulted in a reduction in thermal and visceral nociception. However, the effects of OXA were reversed by the selective OX1R antagonist, SB-334867, which on its own demonstrated hyperalgesic activity [25]. Additionally, Yamamoto et al. demonstrated the analgesic effects of OXA in hot plate and formalin tests, which were suppressed by pretreatment with SB-334867 [58].

The presence of OXRs in the raphe nuclei, the locus coeruleus, and the reticular formation strongly indicates the involvement of the orexinergic system in the regulation of both anxiety-like and nociceptive behaviors [59, 60]. Activation of the OX1R has been shown to increase anxiety-like behaviors induced by orofacial nociception in rats [48, 61]. However, Shahsavari et al. reported that microinjection of OXA into the RVM prevented capsaicin-induced anxiogenic-like behaviors, and these effects were blocked by SB-334867 [62]. The exact mechanisms underlying the contradictory effects of OXA on nociception-induced anxiety necessitate further investigation, and it is plausible that different neuronal pathways and signaling molecules may be implicated, depending on the site of OXA injection. Additional studies are required to delve deeper into these hypotheses and gain a comprehensive understanding of the complex interactions between the orexinergic system, anxiety, and nociceptive responses in the context of TMD.

In our study, we observed that CFA-induced reductions in food and water consumption were mitigated by i.c.v. administration of orexin, an effect that was counteracted by pre-treatment with the OX1R antagonist SB-334867. These findings align with previous research demonstrating that central administration of OXA increases food consumption in rats [21, 63]. Conversely, treatment with SB-334867 has been shown to reduce food intake and alter feeding patterns, leading to weight loss [33, 34]. It is hypothesized that OXA primarily stimulates feeding through OX1 receptors. The orexinergic system plays a crucial role in the hormonal and neural control of energy balance and feeding behavior. Orexin fibers are responsive to glucose levels, with elevated glucose and leptin concentrations suppressing orexinergic neuron activity [64, 65]. Conversely, low glucose or ghrelin levels activate orexin neurons [66, 67]. Additionally, food deprivation has been linked to increased expression of prepro-orexin in the hypothalamus, as well as elevated OX1R and OX2R mRNA levels in various brain regions [68, 69].

In our study, we found that SB-334867 at a concentration of 40 nM effectively prevented OXA responses. It is important to note that OXA exhibits comparable affinity to both OX1R and OX2R, while OXB demonstrates a higher affinity for OX2R over OX1R [70]. SB-334867 inhibits OXRs in a concentration-dependent manner in rats. Indeed, with nanomolar affinity, SB-334867 binds to recombinant human OX1R and effectively blocks the OX1R-mediated calcium response at similar concentrations. Additionally, at higher concentrations, SB-334867 also demonstrates the ability to inhibit the OX2R-mediated calcium response [71, 72].

This study did not explore the specific brain regions where OXA and its antagonist exert their effects. However, it is known that OX1R is widely distributed throughout the brain, with notable expression in hypothalamic nuclei and brain regions involved in the modulation of nociceptive signals, such as the PAG, RVM, and trigeminal spinal nuclei [30, 73]. Previous studies have shown that infusion of OXA into these areas can influence nociceptive and anxiety-related behaviors in rats [28, 35, 62]. Therefore, it is reasonable to hypothesize that central administration of OXA may modulate sensory neuronal networks and molecular signaling in specific brain regions involved in nociceptive processing. However, further experiments are necessary to elucidate the precise mechanisms and intentions of OXA in these brain regions. Understanding the site-specific actions of OXA could provide valuable insights into its therapeutic potential for managing TMD-induced nociception and anxiety-like behaviors.

This study encountered some limitations that should be acknowledged. Firstly, this study focused exclusively on male offspring, potentially overlooking sex-related differences in brain development and nociceptive perception. It is well-established that males and females can exhibit distinct neurobehavioral responses and given the higher prevalence of TMD in females, additional research using female rats is warranted to elucidate the roles of OX-1R in modulating TMD nociception and feeding behavior. Such investigations may shed light on sex-specific differences in the recruitment of autonomic reflexes and endogenous nociception control circuits relevant to TMJ nociception. Furthermore, this study did not measure OXA levels in the control and TMJ noxious stimulation conditions, which would have been valuable in understanding any alterations in orexin signaling in CFA-treated rats. Evaluating OXA levels could provide insights into the potential involvement of the orexinergic system in the pathophysiology of nociceptive responses following CFA administration to the TMJ.

In conclusion, the results of this study suggest that OX1R signaling plays a significant role in modulating CFA-induced TMD nociception, anxiety-like behavior, and feeding abnormalities in rats. However, further experimental investigations are needed to unravel the precise underlying mechanisms through which OX1Rs contribute to these observed effects. Understanding these mechanisms could offer potential therapeutic insights and pave the way for novel approaches in managing TMJ-related nociception.

## Data Availability

Data is available on reasonable request.
